# Editorial: Immunomodulatory natural products - their pharmacological and therapeutic potential

**DOI:** 10.3389/fphar.2026.1843451

**Published:** 2026-04-17

**Authors:** Miao Liu, Jinzhi Duan, Zhuo Luo

**Affiliations:** 1 Department of Pathology, Brigham and Women’s Hospital, Harvard Medical School, Boston, MA, United States; 2 Institute of Health and Medicine, Hefei Comprehensive National Science Center, Hefei, Anhui, China; 3 Guangxi Key Laboratory of Bioactive Molecules Research and Evaluation, College of Pharmacy, Guangxi Medical University, Nanning, China; 4 Guangxi Key Laboratory of Component-Efficacy Relationship of Traditional Chinese Medicine, College of Pharmacy, Guangxi Medical University, Nanning, China

**Keywords:** cancer, immunomodulatory, infectious disease, inflammation, natural product

Natural products continue to attract sustained interest as a source of immunomodulatory agents with broad pharmacological relevance. Their significance extends beyond structural diversity and long-standing empirical use, involving the capacity to regulate immune homeostasis at multiple biological levels, including inflammatory signaling, immune-cell differentiation, tissue microenvironment, host metabolism, and microbiota-associated interactions (Jantan et al., 2015; Zheng et al., 2020; Man et al., 2020). This perspective has gradually replaced the older and less precise view of natural products as generic “immune enhancers.” Increasingly, natural products are being understood as biologically active regulators capable of reshaping disturbed immune states through definable mechanisms and within clearly delimited pathological settings (Jantan et al., 2015; Wainwright et al., 2022).

A major conceptual advance in this area has been the recognition that immunomodulation is seldom a simple matter of stimulation or suppression. Many natural products appear instead to rebalance dysregulated immune networks, with effects that depend on the prevailing biological state. Current evidence increasingly points to multilayer regulation involving immune tolerance, intracellular signaling, and microenvironmental adaptation, especially in pathways related to Treg-centered control of immune homeostasis. Wang et al. Similar patterns have also emerged in oncology, in which improved therapeutic responses are accompanied not only by disease attenuation but also by measurable shifts in immune parameters. Zhang et al. Such observations suggest that the pharmacological value of many natural products lies less in one-directional immune activation than in the restoration of immune equilibrium under pathological conditions (Jantan et al., 2015; Wainwright et al., 2022).

Another important shift has been the movement away from isolated anti-inflammatory observations toward a broader systems-level view of immunopharmacology. Recent studies have linked immunomodulatory activity with coordinated changes spanning immune exhaustion, inflammatory pathways, metabolic disturbance, and microbiota-associated remodeling. Yang et al. A similar expansion in perspective can be seen in chronic inflammatory and allergic disorders, in which symptom improvement is increasingly interpreted alongside broader immunologic readouts and changes in immune-cell behavior. Wang et al. Han et al. This broader framework is important because it moves the field beyond single readouts and toward integrated models that connect immune regulation with disease biology, tissue context, and host response (Zheng et al., 2020; Man et al., 2020).

Host condition has also emerged as a decisive determinant of immunological outcome. Broader research in immunometabolism and microbiota–immune crosstalk has established that immune responses are profoundly conditioned by metabolic and microbial context (Man et al., 2020). This principle is especially relevant to natural products because their effects are often pleiotropic and may depend as much on background physiology as on the intrinsic activity of the intervention itself. In this regard, research indicates that greater systemic metabolic stability is closely linked to a lower risk of severe clinical deterioration, further emphasizing the pivotal role of the host’s metabolic state in shaping immunologically relevant outcomes (Liu et al., 2021). Another study suggested that passive immune-based intervention may produce markedly different effects depending on biological stage and treatment timing, reinforcing the view that immune modulation must be matched to context rather than assumed to be uniformly beneficial (Liu et al., 2020). A broader conceptual framework for passive immune intervention has long emphasized the importance of timing, host status, and mechanism in determining efficacy (Casadevall et al., 2004; Mair-Jenkins et al., 2015). These observations further support a central principle in contemporary immunopharmacology: immune regulation is not a fixed pharmacological property independent of circumstance, but a dynamic process shaped by host state, disease evolution, and mechanistic setting (Man et al., 2020).

The translational implications of this literature are substantial. Natural products are unlikely to fulfill their therapeutic promise if evidence remains limited to variable preparations, incomplete chemical definition, or loosely interpreted biological effects. Progress depends on rigorous source authentication, reproducible composition, careful phytochemical characterization, pharmacokinetic understanding, and direct mechanistic interpretation (Heinrich et al., 2022). These requirements are especially important in natural product research because multicomponent mixtures, metabolism-dependent activity, and host–microbiota interactions can all influence the observed immune phenotype (Zheng et al., 2020; Man et al., 2020). ConPhyMP and related ethnopharmacological best-practice standards have therefore become increasingly important, not merely as reporting tools, but as criteria for deciding whether a claimed effect can truly be regarded as persuasive pharmacological evidence (Heinrich et al., 2022). More disciplined methodology is essential for distinguishing robust immunomodulatory action from attractive but weakly defined biological observations.

Another consequence of this more rigorous framework is a change in therapeutic positioning. The most valuable role of immunomodulatory natural products may not lie in broad stand-alone use, but in reshaping immune environments, improving treatment responsiveness, and complementing established therapeutic strategies in biologically defined settings (Wainwright et al., 2022). Evidence synthesis in oncology has already suggested that synergy may emerge when immune modulation is aligned with disease context and therapeutic background. Zhang et al. Mechanistic analyses centered on immune tolerance and regulatory signaling further support the idea that successful intervention depends on matching the right immune regulator to the right dysfunctional state. Wang et al. Work integrating immune activity with metabolic and microbiota-associated remodeling extends the same logic to complex inflammatory conditions. Yang et al. This direction is more consistent with current pharmacology than older tonic-style narratives because it treats immune regulation as a dynamic, context-bound process rather than a universal property.

Natural products are thus increasingly being understood as modulators of immune equilibrium, disease progression, and therapeutic responsiveness rather than as vaguely defined traditional remedies (Jantan et al., 2015; Wainwright et al., 2022). Continued progress will depend on tighter integration of immunology, chemistry, pharmacology, microbiology, and clinically relevant experimental systems (Wainwright et al., 2022; Heinrich et al., 2022). A more rigorous framework of this kind may help transform immunomodulatory natural products from a broad descriptive category into a more precise therapeutic and conceptual platform for restoring immune balance across complex human disease.
